# Quality of Soybean Products in Terms of Essential Amino Acids Composition

**DOI:** 10.3390/molecules26165071

**Published:** 2021-08-21

**Authors:** Wanda Kudełka, Małgorzata Kowalska, Marzena Popis

**Affiliations:** 1 Department of Quality Food Products, Cracov University of Economics, 31-510 Cracow, Poland; marzena_popis@poczta.onet.pl; 2 Department of Management and Product Quality, Faculty of Chemical Engineering and Commodity Science, Kazimierz Pulaski University of Technology and Humanities, 26-600 Radom, Poland

**Keywords:** soybean, genetically modified soybean, soy products exogenous amino acids

## Abstract

The content of protein, moisture content and essential amino acids in conventional and genetically modified soybean grain and selected soybean products (soybean pâté, soybean drink, soybean dessert, tofu) was analyzed in this paper. The following comparative analysis of these products has not yet been carried out. No differences were observed in the amino acid profiles of soybeans and soybean products. The presence of essential amino acids was confirmed except for tryptophan. Its absence, however, may be due not to its absence in the raw material, but to its decomposition as a result of the acid hydrolysis of the sample occurring during its preparation for amino acid determination. Regardless of the type of soybean grain, the content of protein, moisture content and essential amino acids was similar (statistically insignificant difference). Thus, the type of raw material did not determine these parameters. There was a significant imbalance in the quantitative composition of essential amino acids in individual soybean products. Only statistically significant variation was found in genetically modified and conventional soybean pâté. Moreover, in each soy product their amount was lower irrespective of the raw material from which they were manufactured. Therefore, the authors indicate the necessity of enriching soybean products with complete protein to increase their nutritional value.

## 1. Introduction

Soybean is one of the most important cultivated plants world-wide. Native to East Asia, since the 18th century it has been grown in various regions of the world. Now, most soybeans are grown in North and South America, and in Asia. The reason thereof is the increasing demand for its grain based on the wide-ranging spectrum of its use [1,2,3,4,5]. In addition to the conventional crops, the acreage of genetically modified (GM) soybean crop has also increased steadily. In 2018, the total global area of soybeans was 95.9 million ha and of that 78% was GM soybeans [6,7].

Soybeans are used to produce food products (i.e., soybean oil, tofu/bean curd), food additives (e.g., soy lecithin) and industrial additives, e.g., in the production of cosmetics, plastics, and dyes [1,4,5]. Over the ages, soybean grain and their products, such as soy milk, soya sauce, miso, tofu, and tempeh, have been an important component of the diet in Asian countries. Based on them, pastes and spreads are made as are substitutes of cereal products (bread, pasta, flour), dairy products (soy milk, cheese), or meat substituted [2,8,9,10,11]. Soy milk is a basic ingredient in the production of diverse soy products including tofu, soy yogurt, and cheeses [12,13,14]. On the present market, there are many soy products available and the interest in them constantly grows.

Soybeans are a valuable source of highly nutritional protein, fiber, vitamins, and minerals; fat in the soybeans contains indispensable unsaturated fatty acids [2,15,16]. The soybean proteins are, first of all, globulins that constitute ca. 70% of all the proteins and have auxiliary functions. Other proteins belong to a group of albumins that have enzymatic and structural functions. The majority of them are capable of forming inactive complexes, which impact the biological value and technological usefulness of raw materials for food production; also, they are proteolytic activity regulators. Protease inhibitors constitute ca. 6% of soluble soybean proteins [1,2].

The quality of soybean protein can be compared to proteins of meat, milk, and eggs. Of the plant-based protein sources, the soybean protein is considered to have the highest biological value [17]. The amino acid composition of soybean proteins is close to that of animal protein, in particular the content of exogenous amino acids, i.e., phenylalanine, methionine, threonine, valine, isoleucine, leucine, tryptophan, and lysine. In the reference literature [1,2,17,18], the following percent rates are reported of their contents in the soybean grain: leucine ca. 8 g/100 g of the protein; lysine ca. 6.5 g/100 g of protein; valine ca. 5 g/100 g of the protein; isoleucine ca. 5 g/100 g of the protein; and phenylalanine ca. 4 g/100 g of the protein. Compared with the animal proteins, the soybean protein is characterized by a lower content of sulfur amino acids [18].

In the reference literature, there is a research gap in the detailed knowledge of the nutritional value of conventional and GM soybeans and food products, especially of their amino acid composition [17]. Thus, the objective of the research study was to comparatively analyze the contents of exogenous amino acids in the grain of the conventional and GM soybeans, and in some selected soybean products.

In order to achieve the objective of the research study, the authors assumed the following research hypotheses:

**Hypothesis** **1** **(H1).** *The GM soybean grain and conventional soybean grain contain comparable amounts of exogenous amino acid.*

**Hypothesis** **2** **(H2).** *The type of the raw material used does not decide on the content of exogenous amino acids in the products.*

## 2. Materials and Methods

### 2.1. Material

The research material comprised grain of conventional and GM soybeans, and some selected soy products made from those grains. The GM soy products analyzed consisted of a soy pâté, a soy drink, and a dessert; a soy pâté and a tofu were the conventional soy products for analysis. The research material (both the raw material and the processed soy products) were supplied by soy product manufacturing food plants located in Central Poland.

### 2.2. Methods

The grains of the two types of soybeans were analyzed to determine the type of amino acids present therein, as were the ready-made products from grains of the two types of soybeans. The latter were taken for the analyses after the entire production process accomplished and as soon as the products were packed in individual unit packages. For the purpose of obtaining the repeatability of the analysis results, the analyses were repeated four times and every time different batches of the same product from the same food plant were analyzed.

#### 2.2.1. Determination of Protein

A Kjeldahl method was applied to determine the content of protein in the analyzed GM and conventional soybean grains, and in the soy products. At the first step of the analysis, the content of nitrogen was determined; next, the content of protein was established by multiplying the value of the nitrogen content by 6.25, a standard nitrogen-to-protein conversion factor [19].

#### 2.2.2. Determination of Type and Content of Amino Acids

In order to determine the composition and type of amino acids in the samples analyzed, a hydrolysis process was conducted at first, according to a procedure as described by Spackman et al. [20]. A 24 h hydrolysis was performed using a hydrochloric acid 6 M (6M HCl) with 0.5% of phenol added, under the anaerobic conditions and at a temperature of 110 °C. Methionine was determined by a 16 h acid hydrolysis at 4 °C. Prior to the hydrolysis process, methionine was converted to methionine sulphone resistant to a long-lasting acid hydrolysis (reaction with performic acid) [21]. The content of amino acids was determined using an ion-exchange chromatography (in a 0.37 × 4.5 mm cation-exchange column) with a post-column derivatization and spectrophotometric detection of ninhydryn reaction products, and with the use of an automatic amino acid analyzer (AAA 400, Ingos, Prague, Czech Republic), according to the producer’s standard procedure [22,23].

#### 2.2.3. Determination of Moisture Content

The determination of the moisture content of the grain and the products was performed according to the procedures [6,19]. Drying of the samples was carried out at 103 °C for 24 h.

#### 2.2.4. Statistical Analysis

The calculations were performed with the application of selected procedures of Statistica™10 computer software. A Student’s *t*-test was performed for independent samples in order to compare the content levels of amino acids in the grains of conventional and GM soybeans, and in the products made from them. There was rejected the H_o_:µ_1_ = µ_2_ null-hypothesis having no differences between the level of the feature of the raw material or of the product from conventional soybeans and those from GM soybeans because the calculated value of t-Student’s test was higher that the theoretical value at the selected significance level of α = 0.05.

## 3. Results and Discussion

The protein content in the transgenic grain of soybean was slightly lower (33.01–36.77%) than that in the conventional grain (35.34–37.21%). Those differences were statistically insignificant (Table 1). Compared to the values obtained by the authors of the paper, Garcia et al. [17] determined considerably lower values (23.4%) in the whole soybean grains. Then again, Bohn et al. [6] reported a content value of this component akin to that obtained by the authors of the paper in their research. Additionally, the latter showed a statistically insignificant difference between the protein content in the grains of GM and conventional soybeans (respectively: 34.6% and 34.3%).

Based on the conducted research analyses of the protein content in the soy pâté from GM and conventional soybeans, statistically significant differences were confirmed between those two content levels (Table 1). The soy pâté from GM soybeans had statistically significantly less (6.21–6.57%) protein than that from conventional soybeans (8.10–9.62%). According to Winiarska-Mieczan and Koczmara [24], those differences could be attributed to errors in the technological process. Those authors consider a prolonged time of soybean soaking to be such an error since it might cause explicit losses of the protein content. In addition, they report a slight increase (4%) in the total protein content in dry matter of the 24 h soaked soybean grains. Gallardo et al. [25] believe that it could be caused by the fact that some easily hydrolysing components dissolve and migrate into water. Moreover, they point out a definetely increased activity of enzymes, for example of lipase. Enzymes are proteins, their activity declines, where there is no water, and increases along with the increase in the humidity of the environment; thus, a certain minimum amount of water is indispensable for the enzyme to properly and effectively function. Water imparts conformational plasticity to the enzyme molecule [26]. Conversely, Polthanee and Treloges [27] point out that a long grain soaking time (9 h) might cause the quality of seeds to decrease because of possible adverse microbiological changes. Winiarska-Mieczan and Koczmara [24] inform that the prolongation of the soybean grain soaking time of up to 2 days caused a rapid 13.5% decrease in the protein concentration in soybean grains compared to those soaked only for one day. After 2 days of soaking grains, the content of protein therein was significantly lower than that in the raw grains.

On the whole, the amino acids in foodstuffs undergo changes during processing procedures [2,17]. Amino acids can be precursors of amines in food, which are of a very vital effect on the food quality since they participate in forming a typical, ripe or stale smell of many food products [2,17].

The nutritional quality of protein in legumes, and primarily in soybean, is very high for the composition of that protein is close to that of animal protein [1,17,18]. Table 2 presents the profile and content of exogenous amino acids in the protein of the soybean grains.

Based on the analysis of the amino acid composition of the protein of two types of soybean grains, it was confirmed that, except for tryptophan, exogenous amino acids occurred in that protein (Table 2). The reason for the absence of tryptophan may be due to its degradation during acid hydrolysis [28,29,30]. Among the essential amino acids, leucine and lysine were dominant for both proteins. Methionine was reported to have the lowest content value in the protein of the GM soybean, and it was absent in the profile of exogenous amino acids of the protein of conventional soybean grains.

The type of raw material did not statistically significantly differentiate particular amino acids in the protein of grains of conventional and GM soybean; however, it was found that the amount of all the amino acids was slightly lower in the protein of GM soybean grains (Table 2) and this could be a result of a lower content of protein in this type of soybean (Table 1).

The research conducted by Bøhn et al. [6] just as the research described in this article, did not show any significant differentiation of the content of exogenous amino acids in the grain of conventional and GM soybean. In addition, those authors proved that leucine and lysine were the predominant exogenous amino acids. However, regardless of the type of grain, the content of leucine, valine, isoleucine, and histidine was lower than that reported in this paper. The same authors proved that methionine was also present in both the conventional and GM grain. The content thereof was, respectively: 0.40 g/100 g and 0.42 g/100 g. As regards the content of amino acids in the conventional soybean grain, the results obtained by the authors of this paper are the same as those obtained by Kunachowicz et al. [31]. The only differences between our research results and those by Kunachowicz et al. [31] were that the methionine level as reported in their research was 0.429 g/100 g and the level of phenylalanine was lower. Then, Chen et al. [2] analyzed the amino acid composition of the soybean grain and reported a significantly higher amount of exogenous amino acid compared to the amount as determined in our research. The only exception was histidine. Table 3 shows the determination results of the content and type of amino acids in a ready-to-eat product (soy pâté).

Exogenous amino acids were reported to occur in the protein of the soy pâté from the two soybean types just like in the protein of the grain of the two soybean types. The authors’ own analyses did not confirm only the occurrence of tryptophane just as in the case of the two types of soybean grain. Of exogenous amino acids in the protein of the soy pâté from the conventional soybean grain, leucine, and lysine were present in the highest amounts. The content of methionine was reported to be the lowest (Table 3). Based on the statistical analysis performed, it was concluded that, except for valine, the type of raw material differentiated statistically significantly the contents of exogenous amino acids of the protein in the soy pâté. The amount of amino acids was statistically significantly lower in the protein of the soy pâté from GM soybean grains compared to the product made from the conventional soybean grains (Table 3). The correlation of this kind can be attributed to the fact that the total content of protein in GM soybean grain is lower (Table 1).

The content of exogenous amino acids was statistically significantly lower in the two types of the soy pâté compared to their amounts determined in the raw material from which the soy pâté was produced. This observation confirms that the processing and production procedure of the soy pâté adversely affects the biological value of the protein contained in this product [32,33].

Table 4 shows the amino acid composition of the protein of the soy drink and soy dessert made from GM soybean and, also, of the tofu from conventional soybean.

The analysis results of the amino acid composition of the soy protein in the soy drink, soy dessert and tofu confirmed the occurrence of exogenous amino acids except for tryptophan. However, Rivas et al. [34] confirmed the presence of all the exogenous amino acids in the soy drink. Leucine and lysine were the prevalent exogenous amino acids in the protein of the soy desserts and tofu, while in the protein of the soy drink leucine and isoleucine were most prevalent (Table 4).

The analysis performed shows that no statistically significant differences were reported between the contents of particular amino acids in the protein of the soy drink and soy dessert made from GM soybean grains (Table 4). However, a statistically more significant difference was determined in the soy tofu curd from conventional soybean grains, which is to be attributed to a higher amount of total protein in those products (Table 1) and, also, to a diversified technological process [17]. The latter claim that in the liquid and semi-liquid soy products (respectively: soy drink and soy shake, and soy yoghurt), there are always lower amounts of protein than in the soybean grains; thus, the amount of amino acids therein is lower.

The content of amino acids in the soy products analyzed was statistically significantly lower than that in the raw material. According to Zaremba [30] and Hoffman et al. [32], the technological processes of manufacturing food from soybean grains cause the contents of individual components to decrease several times. Key et al. [35] and Craig [36] claim that adding nutrients to those soy products could prove practical to offset their nutritional value and make it comparable to the nutritional value of the animal protein-based products.

The moisture content of the tested soyabean grains and products containing soybean (soy drink and soy dessert made from GM soybean and tofu from conventional soybean) is summarized in Table 5. 

Based on the results obtained, moisture content was slightly higher in transgenic soybean than in conventional soybean. However, these differences, as in the case of protein content determination, were not statistically significant (Table 5). Other results were reported by Bohn et al. [6], who determined a lower water content of 10.6% in GM soybeans while it was 11.9% in conventional soybeans. On the other hand, Chen et al. [2] reported a content of 8.54% for conventional soybean grain, while Osundahunsi et al. [37] reported 8.4%. Işik E [38] showed that the water content of soybean grains ranged from 10.62–27.06% depending on grain size, while Cai et al. [39] showed 6.20–8.70%. Based on statistical analysis, it was found that the water content of soybean pâté was statistically significantly higher in the product made from conventional grain than in the product made from genetically modified grain (Table 5).

On the other hand, in the soy beverage with genetically modified soybeans, the water content was found to be lower than reported by Osundahunsi et al. [37], 89.6%. Similar values to those reported in the present work are reported by other authors [40,41,42]. Deshpande et al. [40] gave the content of this determinant in soy beverage to be 90.2%, and Cruz et al. [41] found a range between the following values 90–92%. A slightly lower content was determined by Osman and Razig [42], 87%.

Dey et al. [43], found that the water content depends on the type of tofu and can range from 72.0 to 86.8%, which is higher than the value obtained in the presented work. In contrast, Chumchuere et al. [44] determined a water content of 62% in the same product.

## 4. Conclusions

The type of soybean grain had no effect on protein and water content in the product. Only in the case of soybean pâté there was a statistically significant difference in the value of these parameters, which may result from the application of different technological process. Based on the research analyses performed, it was confirmed that, except for tryptophan, exogenous amino acids were present in the soybean grain and in their products. The highest amount levels of exogenous amino acids were determined in the soybean grains regardless of the origin of the raw material. This confirms that the nutritional value for amino acids does not change depending on the grain type. However, in the individual soy products, the contents of exogenous amino acids varied. Those discrepancies could be probably attributed to diverse processing procedures used to produce a given soy product.

## Figures and Tables

**Table 1:** Protein content in soybean grain and soy products.

Product	Raw Material	Content of Protein (g/100 g)			t	p_gran_
		x_min_–x_max_	$\bar{x}$	S_x_ (+/−)		
Grain	GM	33.01–36.77	35.76	0.846	−1.05	0.336
	C	35.34–37.21	36.42	0.942		
Soy pâté	GM	6.21–6.57	6.35	0.156	−6.53 *	0.001
	C	8.10–9.62	8.91	0.769		
Soy drink	GM	2.62–3.00	2.81	0.208	-	-
Soy dessert	GM	3.02–3.47	3.25	0.212		
Tofu	C	12.49–14.63	13.11	1.026		

Symbol * means statistically significant differences at α = 0.05 (p_gran_ < α), C—conventional raw material; GM—raw material.

**Table 2:** Profile and content of exogenous amino acids in conventional and GM soybean grain.

Amino Acids	Grain	Content (g/100 g)		t	p_gran_
		$\bar{x}$	S_x_ (+/−)		
Phenylalanine	GM	1.831	0.091	−2.06	0.085
	C	1.929	0.029		
Leucine	GM	2.762	0.133	−1.09	0.319
	C	2.841	0.059		
Isoleucine	GM	1.662	0.080	−0.91	0.399
	C	1.709	0.066		
Methionine	GM	0.633	0.014	-	-
	C	0.000	0.000		
Valine	GM	1.696	0.102	−0.49	0.643
	C	1.734	0.122		
Lysine	GM	2.288	0.126	−1.14	0.296
	C	2.363	0.039		
Threonine	GM	1.332	0.056	−1.05	0.333
	C	1.382	0.076		
Histidine	GM	1.149	0.067	−0.04	0.968
	C	1.151	0.029		

C—conventional raw material; GM—raw material.

**Table 3:** Profile and content of exogenous amino acids in soy pâté made from conventional and GM soybean grains.

Amino Acids	Soy Pâté	Content (g/100 g)		t	p_gran_
		$\bar{x}$	S_x_ (+/−)		
Phenylalanine	GM	0.295	0.008	−17.76 *	0.000
	C	0.491	0.020		
Leucine	GM	0.457	0.020	−10.00 *	0.000
	C	0.739	0.053		
Isoleucine	GM	0.282	0.003	−10.49 *	0.000
	C	0.451	0.032		
Methionine	GM	0.083	0.006	−4.28 *	0.005
	C	0.112	0.013		
Valine	GM	0.277	0.022	−1.01	0.353
	C	0.444	0.026		
Lysine	GM	0.358	0.009	−10.53 *	0.000
	C	0.554	0.036		
Threonine	GM	0.223	0.021	−9.27 *	0.000
	C	0.352	0.019		
Histidine	GM	0.211	0.014	−3.98 *	0.007

Symbol * means statistically significant differences at α = 0.05 (p_gran_ < α), C—conventional raw material; GM—raw material.

**Table 4:** Profile and content of exogenous amino acids of protein in soy drink and tofu made on the basis of GM soybean grains, and in tofu curd made on the basis of conventional soybean grains.

Amino Acids	Product	Product from Soybean Grain	Content (g/100 g)	
			$\bar{x}$	S_x_ (+/−)
Phenylalanine	Soy drink	GM	0.151	0.001
	Soy dessert		0.157	0.009
	Tofu curd	C	0.767	0.055
Leucine	Soy drink	GM	0.228	0.001
	Soy dessert		0.234	0.004
	Tofu curd	C	1.139	0.070
Isoleucine	Soy drink	GM	0.207	0.136
	Soy dessert		0.145	0.002
	Tofu curd	C	0.689	0.048
Methionine	Soy drink	GM	0.040	0.002
	Soy dessert		0.042	0.002
	Tofu curd	C	0.202	0.004
Valine	Soy drink	GM	0.137	0.001
	Soy dessert		0.141	0.007
	Tofu curd	C	0.680	0.047
Lisine	Soy drink	GM	0.189	0.001
	Soy dessert		0.194	0.005
	Tofu curd	C	0.886	0.054
Threonine	Soy drink	GM	0.105	0.000
	Soy dessert		0.104	0.003
	Tofu curd	C	0.512	0.037
Histidine	Soy drink	GM	0.092	0.001
	Soy dessert		0.093	0.002
	Tofu curd	C	0.409	0.052

C—conventional raw material; GM—raw material.

**Table 5:** Moisture content in soybean grain and soy products.

Product	Raw Material	Moisture Content (g/100 g)		t	p_gran_
		$\bar{x}$	S_x_ (+/−)		
Grain	GM	11.30	0.344	−1.16	0.289
	C	10.90	0.590		
Soy pâté	GM	63.73	1.513	6.22 *	0.001
	C	69.12	0.843		
Soy drink	GM	92.16	0.087	-	-
Soy dessert	GM	81.30	2.391		
Tofu	C	65.67	0.938		

Symbol * means statistically significant differences at α = 0.05 (p_gran_ < α), C—conventional raw material; GM—raw material.

## Data Availability

Not applicable.

## Sample Availability

Samples of the used compounds are available from the authors.
